# A widespread family of polymorphic toxins encoded by temperate phages

**DOI:** 10.1186/s12915-017-0415-1

**Published:** 2017-08-29

**Authors:** Anne Jamet, Marie Touchon, Bruno Ribeiro-Gonçalves, João André Carriço, Alain Charbit, Xavier Nassif, Mario Ramirez, Eduardo P. C. Rocha

**Affiliations:** 1 Instituto de Medicina Molecular, Instituto de Microbiologia, Faculdade de Medicina, Universidade de Lisboa, Lisboa, 1649-028 Portugal; 2 Institut Necker Enfants-Malades, 14 rue Maria Helena Vieira Da Silva, Paris, 75014 France; 3 Université Paris Descartes; Sorbonne Paris Cité, Faculté de Médecine, Paris, 75006 France; 4 INSERM, U1151, Paris, France; 5 CNRS UMR 8253, Paris, France; 6 Microbial Evolutionary Genomics, Institut Pasteur, Paris, 75015 France; 7 CNRS, UMR 3525, Paris, 75015 France

**Keywords:** Bacterial genomics, Phages, Bacterial toxins

## Abstract

**Background:**

Polymorphic toxins (PTs) are multi-domain bacterial exotoxins belonging to distinct families that share common features in terms of domain organization. PTs are found in all major bacterial clades, including many toxic effectors of type V and type VI secretion systems. PTs modulate the dynamics of microbial communities by killing or inhibiting the growth of bacterial competitors lacking protective immunity proteins.

**Results:**

In this work, we identified a novel widespread family of PTs, named MuF toxins, which were exclusively encoded within temperate phages and their prophages. By analyzing the predicted proteomes of 1845 bacteriophages and 2464 bacterial genomes, we found that MuF-containing proteins were frequently part of the DNA packaging module of tailed phages. Interestingly, MuF toxins were abundant in the human gut microbiome.

**Conclusions:**

Our results uncovered the presence of the MuF toxin family in the temperate phages of Firmicutes. The MuF toxin family is likely to play an important role in the ecology of the human microbiota where pathogens and commensal species belonging to the Firmicutes are abundant. We propose that MuF toxins could be delivered by phages into host bacteria and either influence the lysogeny decision or serve as bacterial weapons by inhibiting the growth of competing bacteria.

**Electronic supplementary material:**

The online version of this article (doi:10.1186/s12915-017-0415-1) contains supplementary material, which is available to authorized users.

## Background

Polymorphic toxins (PTs) are multi-domain proteins involved in competition between bacteria and in pathogenesis [1]. Most lineages of bacteria encode at least one PT system in their genome [2]. PTs encompass colicins, soluble pyocins, toxic effectors of type V secretion systems (T5SSs), some toxic effectors of type VI secretion systems (T6SSs), and MafB toxins [1, 3–5]. A PT family is defined by an N-terminal region harboring one or more conserved domains. For instance, a domain of unknown function named DUF1020 (PF06255) is found at the N-termini of all MafB toxins [5], whereas phage late control gene D protein (Phage_GPD; PF05954) and phage base plate assembly protein (Phage_base_V; PF04717) domains are found at the N-termini of VgrG toxins [6]. In contrast, the C-terminus of PTs harbors a set of diverse C-terminal toxin domains, which can have homologs in other distinct PT families [1]. This shared pool of toxin domains between families is a hallmark of PT systems, with more than 150 distinct toxin domains identified so far [2]. Most PTs have RNase, DNase, peptidase, or other protein-modifying activities [2]. PTs can be secreted by various secretion systems. CdiA and BcpA toxins are secreted by CdiB and BcpB, respectively, and belong to the two-partner secretion protein family (*i.e.*, T5SS). CdiA and BcpA toxins require a direct contact for inhibition of bacterial competitors, hence the name contact-dependent inhibition (CDI) system [7, 8]. Certain PT families, including VgrG, Hcp, and PAAR proteins, have structural roles in the T6SS machinery in addition to their toxic activity [9]. In these families, there are two main domain architectures: canonical proteins without a C-terminal extension and PTs bearing a C-terminal extension with toxic activity [6, 10–12]. Besides, it has been demonstrated that a C-terminal extension of a VgrG protein of an enteroaggregative *Escherichia coli* strain did not harbor a toxic domain but mediated binding and transport of a toxic effector [13].

When a bacterium produces an antibacterial toxin, it needs to protect itself from autointoxication and prevent self-inhibition. In most cases a small open reading frame (ORF) encoding a specific immunity protein is located immediately downstream of the toxin gene [14]. PTs modulate the dynamics of microbial communities by killing or inhibiting the growth of competitors lacking the cognate immunity protein. For instance, the predominance of *E. coli* strain EC93 in the intestine of some commercial rats has been linked to CdiA toxin production [15]. Indeed, CdiA of EC93 was shown to inhibit the growth of *E. coli* K12 strains [7]. In *Burkholderia thailandensis*, BcpA is also involved in inhibiting growth of neighboring bacteria through contact [8, 16]. In addition, the Bcp system allows kin discrimination through the immunity proteins produced by bacteria [16]. Furthermore, delivery of BcpA to immune bacteria mediates a contact-dependent signaling that promotes cooperative behavior such as biofilm formation [17]. CdiA and BcpA toxins have only been shown to modulate competitive or cooperative relations between bacteria of the same species. In contrast, effectors of T6SS are involved in both intra- and inter-species competition and can also be delivered into eukaryotic cells [12, 18]. Indeed, a VgrG protein of *Vibrio cholerae* is responsible for remodeling of the actin cytoskeleton when injected into eukaryotic host cells [12], whereas a VgrG toxin of another *Vibrio* strain hydrolyzes the cell wall of Gram-negative competitors [6].

In comparison with the wealth of studies aiming to characterize PT systems in various genera of Proteobacteria (*e*.*g* *.*, *Escherichia, Pseudomonas, Burkholderia, Vibrio*, and *Neisseria*), only the WXG/LXG and Rhs PT families have been studied in monoderms [19, 20]. Monoderms encompass Firmicutes and Actinobacteria and are pivotal in human health. Indeed, they are major constituents of human microbiota accounting for > 50% of the species recovered from skin, nose, stomach, and vagina [21], and they also include major human pathogens [22]. Several WXG/LXG toxins have been described in *Bacillus subtilis* [19, 23], in *Staphylococcus aureus* [24], and in *Streptococcus intermedius* [25]. The role of these toxins in inter-bacterial competition has been demonstrated in the last two species. In addition, an Rhs toxin named WapA confers a competitive advantage in competition assays [20] and is involved in kin discrimination in *B. subtilis* [26].

In this study, we provide an in-depth description of a new family of PTs harboring a domain of the MuF superfamily in their N-terminal region; this family has the unusual feature of being associated with temperate phages. Viruses that infect bacteria (hereafter designated “phages”) may be “virulent,” and thus restricted to act through the lytic cycle, or “temperate.” The latter may either behave like virulent phages or integrate the bacterial chromosomes as prophages. Bacteria harboring prophages are called lysogens and account for nearly half of the sequenced bacteria [27]. Most of the known phages are tailed phages belonging to the *Caudovirales* order [28]. Overall, we found that 35% of the 1753 sequenced tailed phages and 30% of the 2622 prophages harbored a *muf* gene. Among 1515 *muf* genes, 13% encode toxin domains. The presence of a PT system in phages could have important implications for phage biology and microbial population dynamics.

## Results

### The MuF domain-containing proteins constitute a novel polymorphic toxin family

#### Definition of the MuF superfamily of proteins

**Fig. 1:**
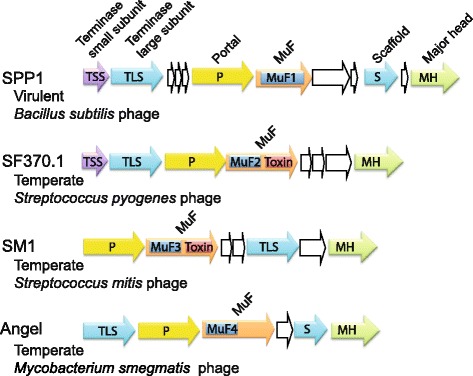
Genetic organization of the head morphogenesis and DNA packaging modules of four phages encoding a MuF protein. SPP1 is a virulent *Bacillus subtilis* phage of the *Siphoviridae* family [31]. SPP1 encodes a short MuF1 protein. SF370.1 is a mitomycin C inducible prophage of M1 serotype *Streptococcus pyogenes* isolate SF370 and belongs to the *Siphoviridae* family [38]. SF370.1 encodes a MuF toxin of the MuF2 family with a putative nuclease activity. SM1 is a mitomycin C inducible prophage of *Streptococcus mitis* belonging to the *Siphoviridae* family [35]. SM1 encodes a MuF toxin of the MuF3 family with a putative nuclease activity. Mycobacteriophage Angel is a temperate phage of *Mycobacterium smegmatis* encoding a MuF4 protein with a C-terminal extension without predicted domain [69]

Mu is a temperate phage infecting *E. coli* which was first discovered due to its striking ability to transpose into the host genome [29]. The name “MuF domain” originated from its original description in protein F of phage Mu (GpF) [30], which is the prototypical MuF protein. Yet, both the MuF domain and the GpF protein have unknown functions. Very few MuF proteins have been characterized so far. The only MuF protein that has been the focus of several experimental studies is Gp7, which is encoded by *Bacillus subtilis* phage SPP1 [31–33]. Both GpF and Gp7 are monodomain proteins harboring a PF04233 domain (Phage_Mu_F domain, hereafter termed MuF1 domain) (Fig. 1). In the National Center for Biotechnology Information (NCBI) Conserved Domain Database, this MuF1 domain is a member of the cl10072 superfamily, which also includes the PF06152 domain (Phage_min_cap2 domain, hereafter termed MuF2 domain). Our initial searches, using hidden Markov model (HMM) profiles of MuF1 and MuF2 domains to retrieve proteins containing a MuF domain, revealed that *muf* genes were located immediately downstream of genes encoding phage portal proteins (Fig. 1), in line with previous observations [2]. We used this contextual genetic information to define two additional MuF domains (MuF3 and MuF4) encoded by genes immediately downstream of phage portal genes and for which the corresponding proteins did not have known domains (see Methods). HMM-HMM comparison [34] of MuF domain HMM profiles showed that MuF3 and MuF4 domains share homology with both PF04233 (MuF1) and PF06152 (MuF2) (Additional file 1: Figure S1). Hence, we enlarged the MuF superfamily by identifying two additional MuF domains (MuF3 and MuF4) for which we built HMM profiles (see Methods and Additional file 2: Table S3).

To detail preliminary indications of a strong association of *muf* genes with phages, we searched for them in bacteriophage and bacterial genomes. We used protein profiles of the four MuF domain families (Additional file 2: Table S3) to retrieve all proteins containing a MuF domain from the predicted proteomes of 1845 bacteriophages and 2464 bacterial genomes (Additional file 2: Tables S1, S2). Altogether, we identified 614 and 901 MuF proteins in bacteriophage genomes and bacterial chromosomes, respectively (Additional file 2: Tables S1, S2, S4, S5). With rare exceptions, phages had only one *muf* gene (614 *muf* genes were retrieved from 611 distinct phage genomes).

#### Domain architecture of MuF domain-containing proteins

**Fig. 2:**
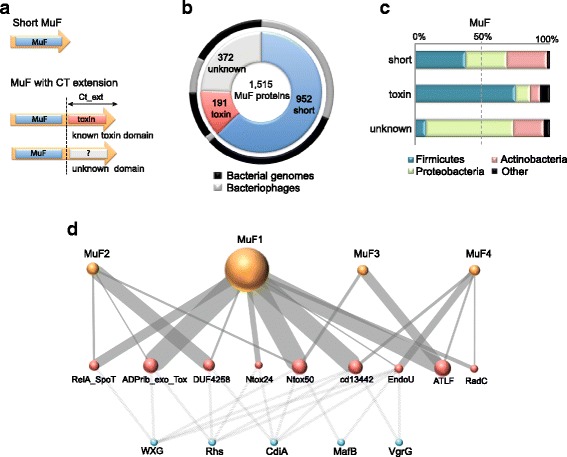
General description of the 1515 MuF proteins detected in this study. **a** Schematic representation of the main domain architectures of MuF proteins. MuF proteins containing a MuF domain (blue box) without a C-terminal extension (Ct_ext) are called short MuFs. MuF proteins with a Ct_ext either harbor a known toxin domain (red box) or an unknown domain (gray box). **b** The inner circle represents the proportion of MuF proteins without Ct_ext (in blue), with a toxin domain (in red), or with an unknown domain (in gray). The outer circle represents their distribution within bacteriophages (in light gray) and bacterial genomes (in black). **c** Taxonomic distribution of bacteria and of hosts of the phages encoding a MuF protein according to the aforementioned categories. There is a significant association of *muf* genes encoding toxin proteins with Firmicutes compared to *muf* genes encoding short proteins (*p* < 0.0001, two-tailed Fisher's exact test), and there is a significant association of *muf* genes encoding proteins with a C-terminal extension without known domain with Proteobacteria compared to *muf* genes encoding short proteins (*p* < 0.0001, two-tailed Fisher's exact test)*.* **d** Association of known toxin domains (red nodes) with MuF domain families (orange nodes) and with other polymorphic toxin families (blue nodes). Only known toxin domains harbored by at least five MuF proteins were reported in this network that includes 172 MuF toxins. The thickness of the edges is proportional to the abundance of the toxin and MuF domain combinations. The size of the orange (MuF families) and red (toxin domains) nodes is proportional to the number of MuF proteins. Toxin domains are described in Additional file 2: Table S7

Among all four families of MuF proteins, we found two major domain architectures: canonical proteins without a C-terminal extension (henceforth called short) and proteins with a C-terminal extension (Fig. 2a). We identified toxin domains among 34% of the latter (Fig. 2b, Additional file 2: Tables S4, S5), corresponding to a total of 13% (*n* = 191) putative toxins among MuF proteins.

As expected for PTs, most toxin domains found among MuF proteins had homologs in other PT families (75%, *e.g*., cd13442, Ntox50, and EndoU_bacteria domains) (Fig. 2d, Additional file 2: Table S7). More than half of the 191 putative MuF toxins had a nuclease domain, and 20% presented a metallopeptidase domain. ADP-ribosyl transferase and RelA-like domains were also frequently identified (Additional file 2: Tables S4, S5, S7).

Intriguingly, phages of Proteobacteria had many MuF proteins harboring a C-terminal extension without known domains (Fig. 2c and Additional file 3: Figure S2). We defined a new domain (termed Ct_MAD for C-terminal MuF Associated Domain) present in one third of these C-terminal extensions (Additional file 2: Tables S3–S5). However, it remains to be determined if the Ct_MAD domain and the remaining C-terminal extensions without known domains correspond to novel toxin domains.

#### Arguments in favor of MuF toxicity

If a bacterium produces a MuF protein with a toxic activity targeting bacterial cytosolic compounds (*i.e*., a nuclease), it should also produce a protective immunity protein to prevent self-intoxication. ORFs encoding immunity proteins are difficult to identify because they are often very short (less than 150 aa) and do not contain known domains [2, 14]. Most (89.4% of the 191 *muf* toxin genes) of the genes encoding MuF toxins identified in our study are followed by a small ORF potentially encoding a polypeptide of less than 150 aa (Additional file 4: Figure S3). In contrast, only 25.5% of the 952 genes encoding short MuF proteins are followed by a similarly small ORF (Additional file 4: Figure S3). MuF toxin genes were more likely than short MuF genes to be associated with small ORFs (*p* < 0.0001, two-tailed Student’s *t* test for the comparison of the lengths of ORFs downstream of *muf* genes) and supports the hypothesis that these small ORFs would encode immunity proteins, necessary only if a toxin domain is present in the MuF protein.

Furthermore, we identified one instance where a MuF toxin C-terminal region together with the small ORF downstream are homologous to a toxin-immunity module of a MafB toxin [5]. As expected, since MuF and Maf are distinct PT systems, the N-terminal regions of the MuF and MafB toxins are unrelated (Additional file 5: Figure S4). Interestingly, this MuF toxin is encoded by a *Streptococcus mitis* phage [35], and the MafB toxin is encoded on a genomic island of a *Neisseria meningitidis* strain. Since both species share the same niche in the human nasopharynx, the possibility of DNA exchange between them may be advocated.

### Distribution of MuF proteins and toxins

#### MuF proteins and toxins are encoded by bacteriophages and bacterial genomes

**Fig. 3:**
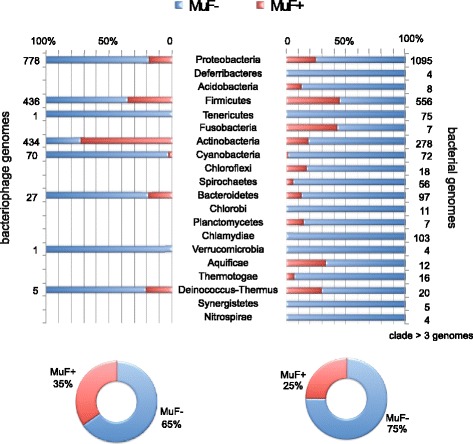
Proportion of genomes encoding MuF per clade. Proportion of genomes encoding at least one MuF protein (*MuF+*, in red) or without MuF protein (*MuF–*, in blue) according to the taxonomy of the host’s *Caudovirales* bacteriophage (left) and of the bacterial genome (right). The total number of genomes in each clade was indicated for both datasets. Only bacterial clades with at least four sequenced genomes were reported (for simplicity). Around 35% of *Caudovirales* and 25% of bacterial genomes contained at least one MuF protein (*MuF+*)

**Fig. 4:**
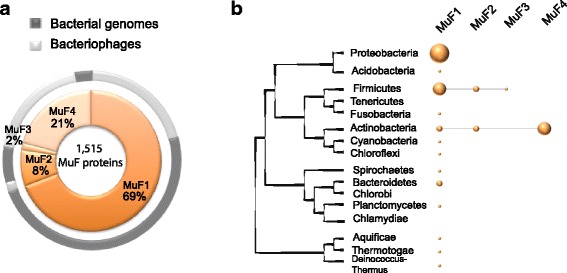
Taxonomic distribution of the 1515 MuF proteins according to their MuF families. **a** Proportion of MuF protein families and their repartitions within bacteriophage (in light gray) and bacterial (in dark gray) genomes. **b** Taxonomic distribution of bacteria and of phages’ hosts encoding MuF proteins. Only bacterial clades with at least eight sequenced genomes were reported (for simplicity). The size of the circles is proportional to the number of MuF proteins

We found that around 35% of the 1753 tailed phages and 25% of the 2464 bacterial genomes in our datasets harbored at least one *muf* gene (Fig. 3). MuF protein families were very unevenly distributed. MuF proteins belonging to the MuF1 family were the most abundant and were identified in many taxa. The three other families were almost exclusively found in Firmicutes and Actinobacteria and their phages (Fig. 4). These results show that MuF domain-containing proteins are widespread. While MuF proteins are equally abundant in Proteobacteria and Firmicutes or their phages, the MuF toxins were much more abundant in Firmicutes or their phages (Fig. 2c and Additional file 3: Figure S2).

The presence of *muf* genes in our bacteriophage dataset led us to investigate in a systematic manner the association of *muf* genes harbored by bacterial genomes with chromosomally integrated phages (*i.e.*, prophages). First, we identified prophages in all bacterial chromosomes (see Methods) and found that 98% of genomes encoding MuF proteins were lysogens. Among the 901 *muf* genes identified in these bacterial chromosomes, most (90%) were located within prophages and 3% within putative prophage remnants (elements smaller than 18 kb; see Methods). Among the remaining, nearly half were found to be located close to genes encoding proteins with similarity to portal or terminase proteins, and could also correspond to prophage remnants (see Methods). Altogether, almost all (96%) of the *muf* genes found in bacterial genomes were associated with either complete prophages or prophage remnants (Additional file 2: Table S6). Reciprocally, around 30% of the 2622 identified prophages (>18 kb) encoded MuF proteins.

#### MuF toxins are associated with temperate tailed phages

**Fig. 5:**
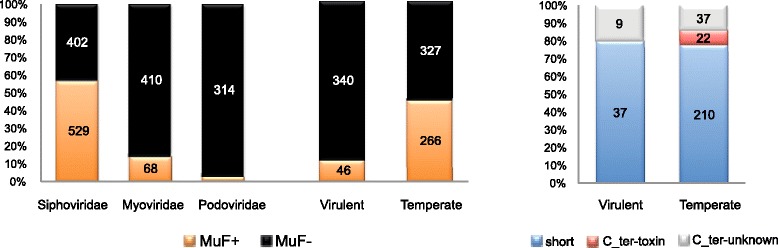
Proportion of bacteriophage genomes encoding MuF per phage family and lifestyle. Proportion of tailed-phage genomes encoding a MuF protein (*MuF+*, in orange) or none (*MuF–*, in black) according to the phage family (left) and the phage lifestyle (center). Proportion of short (in blue), with toxin domain (in red), or with unknown domain (in gray) MuF proteins and their repartition within virulent and temperate tailed phages (right). There is a significant association of *muf* genes with *Siphoviridae* compared to other families of *Caudoviridae* (*p* < 0.0001, two-tailed Fisher's exact test), and there is a significant association of *muf* genes with temperate compared to virulent phages (*p* < 0.0001, two-tailed Fisher's exact test)*.* The association of *muf* toxin genes with temperate phages is not significant (*p* = 0.055, two-tailed Fisher's exact test) due to the small number of *muf* toxin genes in the phage dataset

Local gene organization in bacteria and phages provides important information on the likely function of genes, because genes with closely related functions (partners of a protein complex or enzymes of a pathway) tend to be encoded close in the genome [36, 37]. We thus studied the local genetic context of *muf* genes. In agreement with the genetic context used when defining the MuF superfamily, most (85%) of the 614 *muf* genes identified in bacteriophage genomes had a gene predicted to encode a portal or a terminase domain in the vicinity (Additional file 6: Figure S5A; see Methods). This genetic context around *muf* genes was highly conserved in bacterial chromosomes, since 87% of *muf* genes of this dataset were also detected close to at least one portal-encoding gene or one terminase-encoding gene (Additional file 6: Figure S5). Therefore, *muf* genes were frequently found in the “DNA packaging” module of tailed phages, explaining why they were restricted to the *Caudovirales.* Among the 1753 *Caudovirales* phages of our dataset (Additional file 2: Table S1), 35% encoded a MuF protein. But these were not evenly distributed between the *Caudovirales* families, with most MuF proteins encoded by *Siphoviridae* (86%), 11% by *Myoviridae*, and the remaining by *Podoviridae* (Fig. 5 and Additional file 2: Tables S1–S4). Strikingly, more than half (57%) of the 931 *Siphoviridae* of our dataset encoded a MuF protein (Fig. 5). The abundance of MuF proteins encoded by *Siphoviridae* genomes suggests a general functional or structural role of MuF proteins locating in the viral head of *Caudovirales*, in line with the experimental data on the Gp7 protein of *Bacillus subtilis* bacteriophage SPP1 [31–33]. Indeed, in bacteriophage SPP1, Gp7 binds the portal protein and is present in one to two copies per capsid [31].

We predicted the lifestyle (virulent versus temperate) of 979 *Caudovirales* phages (see Methods, Additional file 2: Table S1), which allowed us to determine that virulent phages encoded very few MuF proteins and contained no single MuF protein with a known toxin domain (Fig. 5). In contrast, many temperate phages infecting Firmicutes encoded MuF proteins with toxin domains (Additional file 3: Figure S2C). In Proteobacteria, MuF proteins with a Ct_MAD domain were also restricted to temperate phages (Additional file 2: Table S4). Since MuF toxins are restricted to temperate phages, and the genes found in bacterial genomes are in prophages, we hypothesized that there is most likely a link between lysogeny and MuF proteins carrying toxin domains.

Among the prophages harboring MuF toxin genes, several have been shown to be inducible, including SF370.1 of *Streptococcus pyogenes* [38], SM1 of *Streptococcus mitis* [35], StB12 of *Staphylococcus hominis* [39], and several prophages of *Enterococcus faecalis* [40]. Hence, MuF toxins are present in fully functional prophages. Furthermore, in a proteomic analysis of the StB12 phage, the MuF toxin was detected [39].

#### Genes encoding MuF toxins are present in the human gut microbiota

Our results on bacteriophages and bacterial genomes demonstrated a strong association of MuF toxins with both temperate phages and prophages of Firmicutes. Hence, these toxins could play an important role in gut microbiota, especially since more than half of the bacteria found in the gut belong to the Firmicutes phylum [21]. We thus searched for MuF proteins in human gut microbiomes, using a non-redundant catalog of 10 million proteins [41]. We found that 51% of the 6406 MuF proteins present in the human gut catalog have a C-terminal extension and 67% of these have known toxin domains (Additional file 2: Table S8). These results strongly favor a role for MuF toxins in the bacterial population ecology of the human gut microbiome by influencing bacteria-phage interactions.

## Discussion

In this work, we discovered many PTs associated with MuF domains on temperate phages. The most studied MuF protein is encoded by *Bacillus subtilis* phage SPP1 (Gp7) [31–33], where it is present in one to two copies per capsid in a complex with portal proteins [31]. Gp7 harbors no toxin domain. To our knowledge, the only MuF protein with a toxin domain studied so far is the EFV toxin (Q838U8) encoded by a lysogenic phage of the *Enterococcus faecalis* strain V583 [42]. EFV toxin is a MuF1 protein with an ADP-ribosyl transferase activity, which is toxic when expressed in yeast [42].

We hypothesize that the toxin domain of MuF proteins could be delivered into host bacteria during phage DNA injection. In contrast to the widespread distribution of *muf* genes, those encoding a toxin domain were present only in temperate phages and were vastly over-represented in prophages of Firmicutes. These findings suggest that MuF toxins could influence the lysogenic decision. Alternatively, MuF toxins could act as molecular weapons in inter-bacterial competition.

There are many examples of prophages encoding toxins with anti-eukaryotic activities involved in bacterial virulence [43]. These include *E. coli* prophages encoding Shiga toxin, filamentous phage CTXφ encoding the cholera toxin, or *S. aureus* prophages encoding the Panton-Valentine leukocidin [43]. Besides, several prophage toxin-antitoxin (TA) genes were reported [44–47], *e.g*., in extra-chromosomal prophages P1 and N15, where they may stabilize the presence of prophages though post-segregational killing, as is often the case in plasmids [48]. In addition, multi-protein structures termed “tailocins” have been described in the genomes of *Pseudomonas*. Tailocins are morphologically similar to phage tails and exhibit a bacteriotoxic activity via direct perforation of the cell envelope. Bacteriocins are often encoded close to the tail cassette [49].

The predicted targets of MuF toxins encompass RNA molecules, for putative ribonucleases, and (p)ppGpp metabolism, for putative RelA-like MuF toxins (Additional file 2: Table S7). Both substrates are likely to be primarily encountered in the host cytoplasm. We hypothesize that MuF toxins are loaded into the viral head to be delivered to bacteria concomitantly with the injection of phage DNA into the host cytoplasm. Infecting phages are known to inject proteins along with their DNA in the bacterial cytoplasm, as demonstrated for several ADP-ribosyl transferase proteins of virulent T4 phage [50]. In support of this hypothesis, 14% of the 191 MuF toxins identified in our study are also putative ADP-ribosyl transferases (Additional file 2: Tables S4, S5).

**Fig. 6:**
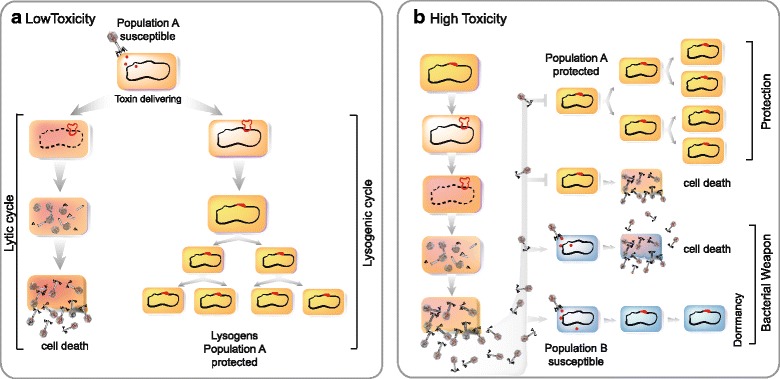
Proposed model for the role of phage-delivered MuF toxins exhibiting a low (**a**) or high (**b**) toxic effect in host bacteria. **a** MuF toxin with a low toxic effect such as proteases or ADP-ribosyl transferases could influence the lysis-lysogeny decision of the phage with the aim of making the optimal decision at the time of infection. **b** MuF toxin with a high toxic effect could be involved in inter-bacterial competition. When population A of lysogens is mixed with population B of non-lysogens, phages carrying a MuF toxin could deliver their toxins to susceptible bacteria of population B. The delivery of a toxin with a highly toxic effect, such as a nuclease, into bacteria of population B leads to either a direct inhibition of their growth (dormancy) or killing by induction of the lytic cycle. In both cases, population A of lysogens will outcompete population B of non-lysogens. Thus, bacteria harboring a phage encoding a MuF toxin will have a competitive advantage

What could be the role of MuF toxins? If these proteins, or their toxin domains, are delivered into the host cytoplasm, their effects may resemble those of homologous toxin domains of T6SS and T5SS, which lead to growth inhibition of the competitors. The presence of MuF toxins almost exclusively on temperate phages and prophages suggests an association with lysogeny. We speculate that the role of MuF toxins will depend on the level of toxicity of the protein. If the few MuF toxin protein molecules carried by the phage are moderately toxic, as expected from peptidase or ADP-ribosyl transferase activities, they could guide the decision between lysis and lysogeny, for instance by sensing growth, or providing resistance to cellular defense mechanisms (Fig. 6a).

Some PTs with nuclease activity are known to be highly toxic and serve as antibacterial weapons. For instance, it has been demonstrated that the cd13442 toxin domain of the CdiA-2 toxin (I1WVY3) of *Burkholderia pseudomallei* 1026b has tRNA nuclease activity and is required for contact-dependent growth inhibition of neighboring competitors [51, 52]. Such highly toxic MuFs could enhance the role of temperate phages in bacterial competition (Fig. 6b). It has been proposed that bacteria use their prophages to remove competitors from a niche [53], and that this could impact bacterial pathogenesis [54]. This mechanism would work as follows: a lysogenic population would produce phages by lysis of a subpopulation of cells and thus kill sensitive competitors (siblings are protected by their own prophages encoding an immunity protein). However, the effect of this mechanism is short-lived, since the phage will eventually lysogenize some competitors, rendering them immune to superinfection [55]. This is where the MuF toxin may play a role. If infection by the phage results in lysogeny, the incoming toxin could, at least temporarily, inhibit bacterial growth and provide a competitive advantage to the population carrying the prophage (Fig. 6b). Hence, phages would either kill or inhibit the growth of the competing population.

## Conclusions

The abundance of MuF PTs suggests the existence of a toxin phage-delivery system and raises intriguing possibilities for their function in the context of phage-bacteria and inter-bacterial interactions. The latter could be important in environments such as human-associated ecosystems, where multiple strains of the same species or closely related species compete for the same niche [56]. Strikingly, MuF toxins are particularly abundant in phages and prophages of Firmicutes that are significant members of the human microbiota in various niches [21] and include major human pathogens. Indeed, we found MuF toxins in important human pathogens including *Streptococcus pyogenes* and *Enterococcus faecalis*.

## Methods

### Datasets

The sequences and corresponding annotations of 1845 complete bacteriophage genomes and 2462 complete bacterial genomes were retrieved from GenBank Refseq (last accessed September 2016) [57].

The phage dataset contained 1753 *Caudovirales* (95%), of which 53%, 27%, and 19% were *Siphoviridae*, *Myoviridae*, and *Podoviridae*, respectively. The order, the family, the type of nucleic acid, and the bacterial host of bacteriophages were extracted from the GenBank files. The lifestyle of *Caudovirales* phages was predicted using Phage Classification Tool Set (PHACTS) v0.3 [58] (as in [27]). PHACTS predicts the lifestyle of a phage (*i.e*., virulent or temperate) from genomic data using both a similarity and a supervised random forest algorithm. The classification is based on the similarity to phages with known lifestyles in a manually curated database. Predictions were considered as confident only if the averaged probability score of the predicted lifestyle is two standard deviations (SDs) away from the averaged probability score of the other lifestyle, as recommended by the authors (who claim a precision rate of 99% with this parameter). We classified with such high confidence 55% of the dataset (*i.e*., 410 virulent and 600 temperate phages). The general characteristics and the result of lifestyle prediction for each phage are listed in Additional file 2: Table S1.

Prophages were detected in bacterial chromosomes as in [27] using Phage Finder v4.6 [59]. The elements larger than 18 kb were considered as prophages. The smaller elements identified by Phage Finder may be prophage remnants or erroneous assignments. Thus, we identified 2622 large prophages (>18 kb). We found that 50% of the strains were lysogenic (*i.e*., contained at least one large prophage), consistent with our previous analysis [27]. The characteristics of the bacterial genomes and their prophages are reported in Additional file 2: Table S2.

Amino acid sequences of the integrated reference catalog of the human gut microbiome were retrieved from [41].

### Construction of MuF and Ct_MAD profiles

The HMM profiles PF04233 corresponding to MuF1 and PF06152 corresponding to MuF2 were retrieved from the Pfam 28.0 database [60] (for details see Additional file 2: Table S3). We built profiles for MuF3 and MuF4. For this, we used N-terminal regions (250 amino acids) of the Gp35 protein from *Streptococcus mitis* phage SM1 (NC_004996) and of the Gp4 protein from mycobacteriophage Angel (NC_012788) as seed sequences for MuF3 and MuF4, respectively. Seed sequences were used as queries for the Position-Specific Iterative (PSI)-Basic Local Alignment Search Tool (BLAST) algorithm of the BLASTP 2.3.1+ program [57] that was used to search homologies in the NCBI's non-redundant (nr) protein database with default parameters. Redundancy of the sequence set retrieved from BLASTP was reduced using CD-HIT v4.6 [61] with a 90% identity threshold. The longest representative sequence of each cluster was then aligned with MUSCLE v3.8.31 [62]. HMM profiles were built from the multiple sequence alignments using *hmmbuild* of HMMER v3.1b1 [63] with default parameters.

To build the HMM profile Ct_MAD, we used the C-terminal extensions of MuF proteins without known domains and clustered them with Single Linkage Clustering of Sequences (SiLiX, sequence identity ≥ 30% and overlap ≥ 50%) [64]. We obtained eight families with more than five sequences. Domain analysis with CDvist tool [65] showed that four of these families have hits within the C-terminal region of COG2369, which is a cluster of orthologous groups including proteins with a MuF1 domain. The profile Ct_MAD was built from the multiple sequence alignment of the sequences of the four families following the same procedure as for MuF3 and MuF4. MuF3, MuF4, and Ct_MAD HMM profiles are available (Additional files 7, 8, and 9).

HMM profiles of the four MuF domains were compared using the HHsearch program of HH-suite v2.0.15 with default parameters [66]. HMM profiles used for comparison were built with the HHmake program of HH-suite from the same seed alignments previously used to build the profiles with HMMER. Cytoscape v3.4.0 was used to visualize the resulting domain association network (Additional file 1: Figure S1) [67].

### Genetic context of genes encoding a MuF domain in bacteriophages and bacterial chromosomes

A *muf* gene was regarded as part of a prophage if it was within the boundaries of the large prophage coordinates identified within bacterial chromosomes by Phage Finder (see above). It was considered as part of a remnant prophage if it was located within prophages smaller than 18 kb or if it was near portal and/or terminase genes.

We retrieved all the HMM profiles known to be associated with portal (P), terminase small (TSS), and large subunit (TLS) proteins from Pfam and TIGRFam (Additional file 2: Table S3). We performed a search of these profiles in bacteriophages and bacterial chromosomes using HMMER3 v3.1b1 with the –cut_ga option. We only selected the best e-value profile for each hit and considered, for each of the three categories, *i.e*., P, TSS, and TSL, the closest gene to the *muf* gene. Then, we computed the minimal distance between each *muf* gene and P, TLS, and TSS genes (10 genes around each *muf* gene). This allowed us to clearly identify the genetic context in the vicinity of *muf* genes in both bacteriophages and bacterial chromosomes.

Since more than 60% of *muf* genes were located in +1 of a P gene, we analyzed proteins encoded by genes immediately downstream of P genes (+1) in the phage dataset in order to search for possible additional MuF domains. We did not identify proteins belonging to additional MuF families among the proteins encoded by these genes.

### Identification of MuF proteins

We used the HMM profiles of MuF1, MuF2, MuF3, and MuF4 domains to scan our datasets of bacteriophages and bacterial genomes using the *hmmsearch* program of HMMER v3.1b1 with the –cut_ga option. The results of the detection and the RefSeq accession numbers of all the MuF proteins are reported for phages and bacterial genomes in Additional file 2: Tables S4 and S5, respectively. The list of the MuF proteins retrieved from the integrated reference catalog of the human gut microbiome is provided in Additional file 2: Table S8.

### Architecture of MuF proteins

Domain architectures of MuF proteins were analyzed with the CDvist tool [65] against the Pfam 28.0 domain database [60] and the Conserved Domain Database (CDD) v3.12 [57]. To determine the minimal length required to define the presence of a C-terminal extension, we searched for the shortest C-terminal extension containing a toxin domain in our dataset. We found a MuF4 toxin with an endoU domain from *Bifidobacterium bifidum* carrying a C-terminal extension of 113 amino acids. Consequently, we set a cut-off equal to or greater than 100 amino acids to define the presence of a C-terminal extension.

The occurrence of MuF-associated toxin domains in other PT families was investigated using the Conserved Domain Architecture Retrieval Tool (CDART) of NCBI [68]. Cytoscape v3.4.0 was used to visualize the resulting domain association network [67].

### Statistical analyses

All statistical analyses were performed in R. A Fisher’s exact test (fisher.test in R) was used to determine whether proportions of one variable between two groups are significantly different. A two-sample unpaired Student’s *t* test (t.test in R) was used to determine statistically significant differences between the mean values on data with two categories.

## Additional files

Additional file 1: Figure S1. Relationships between MuF families. Network association of the four HMM profiles of MuF proteins. Each node corresponds to a MuF HMM profile, and each edge width is proportional to the probability of homologous relationship computed by HHsearch for pairwise comparison of HMM profiles using HH-suite (see Methods). (PDF 381 kb)

Additional file 2: Table S1. General characteristics of bacteriophages, results of the prediction of lifestyle, and of the detection of MuF. **Table S2.** General characteristics of bacterial genomes, prophages and information on whether they encode a MuF protein. **Table S3.** General characteristics of the HMM protein profiles. **Table S4.** General characteristics of the 614 MuF proteins from bacteriophages. **Table S5.** General characteristics of the 901 MuF proteins identified in bacterial genomes. **Table S6.** Information on the bacterial prophages where *muf* genes could be identified. **Table S7.** General characteristics of toxin domains. **Table S8.** List of the MuF proteins identified in the integrated reference catalog of the human gut microbiome [41]. (XLSX 1074 kb)

Additional file 3: Figure S2. Proportion of toxin domains in each MuF family and taxonomical clade. Distribution of MuF protein families in bacteriophages (A) and in bacterial chromosomes (B). The number of MuF proteins detected was reported (in *orange*) for each clade. Proportion of MuF domains associated with a C-terminal extension (Ct_ext) with known toxin domains (*Ct_ext-tox*, in *red*), with unknown domains (*Ct_ext-unk*, in *gray*) or without Ct_ext (*Short*, in *blue*) in bacteriophages (C) and in bacterial chromosomes (D). Only the number of MuF proteins with a known toxin domain, and only bacterial clades with at least four genomes sequenced were indicated (for simplicity). (PDF 832 kb)

Additional file 4: Figure S3. Distribution of the length of the proteins encoded by genes annotated immediately downstream of *muf* genes. The association of small ORFs with MuF toxins compared to short MuFs is highly significant (two-tailed unpaired Student’s *t* test, *p* < 0.0001). The three categories correspond to the MuF protein architecture. *Ct-tox* MuF domains associated with a C-terminal extension with known toxin domains, *Ct-unk* MuF domains associated with a C-terminal extension with unknown domains, *Short* MuF domains without C-terminal extension. (PDF 70 kb)

Additional file 5: Figure S4. Pairwise comparison of the genetic organization of *muf* and *maf* genes. *Top*: the *muf* gene and the downstream ORF found in phage SM1 (NC_004996) infecting *Streptococcus mitis. Bottom*: the *mafB* toxin gene (encoding WP_003711327.1) and the cognate immunity gene *mafI* (encoding WP_002235294.1) found in *Neisseria meningitidis* NM3001 (assembly GCA_000293665.1). Nucleotide comparison was generated using BLASTn implemented in Easyfig 2.1. *Gray vertical block* indicates regions of shared similarity shaded according to BLASTn identity. The level of nucleotide identity is shown in the gradient scale. (PDF 74 kb)

Additional file 6: Figure S5. Genetic context in the vicinity of *muf* genes. Genetic context in the vicinity of *muf* genes detected in bacteriophage genomes (A) and bacterial chromosomes (typically prophages, B). The total number of genes encoding portal (in *yellow*), terminase large subunit (in *blue*), and small subunit (in *purple*) proteins according to their distances to *muf* genes (distance = 0) were reported (see Methods). 85% and 87% of *muf* genes were detected close to at least a portal-encoding gene or a terminase-encoding gene in bacteriophage genomes and bacterial chromosomes, respectively. The genetic context around *muf* genes was highly conserved in both datasets. (PDF 442 kb)

Additional file 7: HMMER3 hidden Markov model for MuF3. (HMM 113 kb)

Additional file 8: HMMER3 hidden Markov model for MuF4. (HMM 107 kb)

Additional file 9: HMMER3 hidden Markov model for C-terminal MuF associated domain Ct_MAD. (HMM 114 kb)

## References

[1] Jamet A, Nassif X (2015). New players in the toxin field: polymorphic toxin systems in bacteria. MBio.

[2] Zhang D, de Souza RF, Anantharaman V, Iyer LM, Aravind L (2012). Polymorphic toxin systems: comprehensive characterization of trafficking modes, processing, mechanisms of action, immunity and ecology using comparative genomics. Biol Direct.

[3] Poole SJ, Diner EJ, Aoki SK, Braaten BA, t'Kint de Roodenbeke C, Low DA, Hayes CS (2011). Identification of functional toxin/immunity genes linked to contact-dependent growth inhibition (CDI) and rearrangement hotspot (Rhs) systems. PLoS Genet.

[4] Ghequire MG, De Mot R (2014). Ribosomally encoded antibacterial proteins and peptides from Pseudomonas. FEMS Microbiol Rev.

[5] Jamet A, Jousset AB, Euphrasie D, Mukorako P, Boucharlat A, Ducousso A, Charbit A, Nassif X (2015). A new family of secreted toxins in pathogenic Neisseria species. PLoS Pathog.

[6] Brooks TM, Unterweger D, Bachmann V, Kostiuk B, Pukatzki S (2013). Lytic activity of the Vibrio cholerae type VI secretion toxin VgrG-3 is inhibited by the antitoxin TsaB. J Biol Chem.

[7] Aoki SK, Pamma R, Hernday AD, Bickham JE, Braaten BA, Low DA (2005). Contact-dependent inhibition of growth in Escherichia coli. Science.

[8] Anderson MS, Garcia EC, Cotter PA (2012). The Burkholderia bcpAIOB genes define unique classes of two-partner secretion and contact dependent growth inhibition systems. PLoS Genet.

[9] Durand E, Cambillau C, Cascales E, Journet L (2014). VgrG, Tae, Tle, and beyond: the versatile arsenal of Type VI secretion effectors. Trends Microbiol.

[10] Shneider MM, Buth SA, Ho BT, Basler M, Mekalanos JJ, Leiman PG (2013). PAAR-repeat proteins sharpen and diversify the type VI secretion system spike. Nature.

[11] Ma J, Pan Z, Huang J, Sun M, Lu C, Yao H. The Hcp proteins fused with diverse extended-toxin domains represent a novel pattern of antibacterial effectors in type VI secretion systems. Virulence. 2017;Jan 6:1–14. doi:10.1080/21505594.2017.1279374. 10.1080/21505594.2017.1279374 PMC5711352 28060574

[12] Pukatzki S, Ma AT, Revel AT, Sturtevant D, Mekalanos JJ (2007). Type VI secretion system translocates a phage tail spike-like protein into target cells where it cross-links actin. Proc Natl Acad Sci U S A.

[13] Flaugnatti N, Le TT, Canaan S, Aschtgen MS, Nguyen VS, Blangy S, Kellenberger C, Roussel A, Cambillau C, Cascales E (2016). A phospholipase A1 antibacterial Type VI secretion effector interacts directly with the C-terminal domain of the VgrG spike protein for delivery. Mol Microbiol.

[14] Zhang D, Iyer LM, Aravind L (2011). A novel immunity system for bacterial nucleic acid degrading toxins and its recruitment in various eukaryotic and DNA viral systems. Nucleic Acids Res.

[15] Aoki SK, Poole SJ, Hayes CS, Low DA (2011). Toxin on a stick: modular CDI toxin delivery systems play roles in bacterial competition. Virulence.

[16] Anderson MS, Garcia EC, Cotter PA (2014). Kind discrimination and competitive exclusion mediated by contact-dependent growth inhibition systems shape biofilm community structure. PLoS Pathog.

[17] Garcia EC, Perault AI, Marlatt SA, Cotter PA (2016). Interbacterial signaling via Burkholderia contact-dependent growth inhibition system proteins. Proc Natl Acad Sci U S A.

[18] Jiang F, Waterfield NR, Yang J, Yang G, Jin Q (2014). A Pseudomonas aeruginosa type VI secretion phospholipase D effector targets both prokaryotic and eukaryotic cells. Cell Host Microbe.

[19] Holberger LE, Garza-Sanchez F, Lamoureux J, Low DA, Hayes CS (2012). A novel family of toxin/antitoxin proteins in Bacillus species. FEBS Lett.

[20] Koskiniemi S, Lamoureux JG, Nikolakakis KC, t'Kint de Roodenbeke C, Kaplan MD, Low DA, Hayes CS (2013). Rhs proteins from diverse bacteria mediate intercellular competition. Proc Natl Acad Sci U S A.

[21] Cho I, Blaser MJ (2012). The human microbiome: at the interface of health and disease. Nat Rev Genet.

[22] Woodford N, Livermore DM (2009). Infections caused by Gram-positive bacteria: a review of the global challenge. J Infect.

[23] Elbaz M, Ben-Yehuda S (2015). Following the fate of bacterial cells experiencing sudden chromosome loss. MBio.

[24] Cao Z, Casabona MG, Kneuper H, Chalmers JD, Palmer T (2016). The type VII secretion system of Staphylococcus aureus secretes a nuclease toxin that targets competitor bacteria. Nat Microbiol.

[25] Whitney JC, Peterson SB, Kim J, Pazos M, Verster AJ, Radey MC, Kulasekara HD, Ching MQ, Bullen NP, Bryant D, et al. A broadly distributed toxin family mediates contact-dependent antagonism between gram-positive bacteria. Elife. 2017;6. 10.7554/eLife.26938 PMC5555719 28696203

[26] Lyons NA, Kraigher B, Stefanic P, Mandic-Mulec I, Kolter R (2016). A combinatorial kin discrimination system in Bacillus subtilis. Curr Biol.

[27] Touchon M, Bernheim A, Rocha EP (2016). Genetic and life-history traits associated with the distribution of prophages in bacteria. ISME J.

[28] Fokine A, Rossmann MG (2014). Molecular architecture of tailed double-stranded DNA phages. Bacteriophage.

[29] Harshey RM (2012). The Mu story: how a maverick phage moved the field forward. Mob DNA.

[30] Giphart-Gassler M, Wijffelman C, Reeve J (1981). Structural polypeptides and products of late genes of bacteriophage Mu: characterization and functional aspects. J Mol Biol.

[31] Droge A, Santos MA, Stiege AC, Alonso JC, Lurz R, Trautner TA, Tavares P (2000). Shape and DNA packaging activity of bacteriophage SPP1 procapsid: protein components and interactions during assembly. J Mol Biol.

[32] Stiege AC, Isidro A, Droge A, Tavares P (2003). Specific targeting of a DNA-binding protein to the SPP1 procapsid by interaction with the portal oligomer. Mol Microbiol.

[33] Vinga I, Droge A, Stiege AC, Lurz R, Santos MA, Daugelavicius R, Tavares P (2006). The minor capsid protein gp7 of bacteriophage SPP1 is required for efficient infection of Bacillus subtilis. Mol Microbiol.

[34] Soding J (2005). Protein homology detection by HMM-HMM comparison. Bioinformatics.

[35] Siboo IR, Bensing BA, Sullam PM (2003). Genomic organization and molecular characterization of SM1, a temperate bacteriophage of Streptococcus mitis. J Bacteriol.

[36] Overbeek R, Fonstein M, D'Souza M, Pusch GD, Maltsev N (1999). The use of gene clusters to infer functional coupling. Proc Natl Acad Sci U S A.

[37] Huynen M, Snel B, Lathe W, Bork P (2000). Predicting protein function by genomic context: quantitative evaluation and qualitative inferences. Genome Res.

[38] Canchaya C, Desiere F, McShan WM, Ferretti JJ, Parkhill J, Brussow H (2002). Genome analysis of an inducible prophage and prophage remnants integrated in the Streptococcus pyogenes strain SF370. Virology.

[39] Deghorain M, Bobay LM, Smeesters PR, Bousbata S, Vermeersch M, Perez-Morga D, Dreze PA, Rocha EP, Touchon M, Van Melderen L (2012). Characterization of novel phages isolated in coagulase-negative staphylococci reveals evolutionary relationships with Staphylococcus aureus phages. J Bacteriol.

[40] Matos RC, Lapaque N, Rigottier-Gois L, Debarbieux L, Meylheuc T, Gonzalez-Zorn B, Repoila F, Lopes Mde F, Serror P (2013). Enterococcus faecalis prophage dynamics and contributions to pathogenic traits. PLoS Genet.

[41] Li J, Jia H, Cai X, Zhong H, Feng Q, Sunagawa S, Arumugam M, Kultima JR, Prifti E, Nielsen T (2014). An integrated catalog of reference genes in the human gut microbiome. Nat Biotechnol.

[42] Fieldhouse RJ, Turgeon Z, White D, Merrill AR (2010). Cholera- and anthrax-like toxins are among several new ADP-ribosyltransferases. PLoS Comput Biol.

[43] Canchaya C, Proux C, Fournous G, Bruttin A, Brussow H. Prophage genomics. Microbiol Mol Biol Rev. 2003;67(2):238–276, table of contents. 10.1128/MMBR.67.2.238-276.2003 PMC156470 12794192

[44] Guo Y, Quiroga C, Chen Q, McAnulty MJ, Benedik MJ, Wood TK, Wang X (2014). RalR (a DNase) and RalA (a small RNA) form a type I toxin-antitoxin system in Escherichia coli. Nucleic Acids Res.

[45] Romero P, Croucher NJ, Hiller NL, Hu FZ, Ehrlich GD, Bentley SD, Garcia E, Mitchell TJ (2009). Comparative genomic analysis of ten Streptococcus pneumoniae temperate bacteriophages. J Bacteriol.

[46] Goeders N, Van Melderen L (2014). Toxin-antitoxin systems as multilevel interaction systems. Toxins (Basel).

[47] Chan WT, Yeo CC, Sadowy E, Espinosa M (2014). Functional validation of putative toxin-antitoxin genes from the Gram-positive pathogen Streptococcus pneumoniae: phd-doc is the fourth bona-fide operon. Front Microbiol.

[48] Van Melderen L, Saavedra De Bast M (2009). Bacterial toxin-antitoxin systems: more than selfish entities?. PLoS Genet.

[49] Ghequire MG, Dillen Y, Lambrichts I, Proost P, Wattiez R, De Mot R (2015). Different ancestries of R tailocins in rhizospheric Pseudomonas isolates. Genome Biol Evol.

[50] Corda D, Di Girolamo M (2003). Functional aspects of protein mono-ADP-ribosylation. EMBO J.

[51] Nikolakakis K, Amber S, Wilbur JS, Diner EJ, Aoki SK, Poole SJ, Tuanyok A, Keim PS, Peacock S, Hayes CS (2012). The toxin/immunity network of Burkholderia pseudomallei contact-dependent growth inhibition (CDI) systems. Mol Microbiol.

[52] Morse RP, Nikolakakis KC, Willett JL, Gerrick E, Low DA, Hayes CS, Goulding CW (2012). Structural basis of toxicity and immunity in contact-dependent growth inhibition (CDI) systems. Proc Natl Acad Sci U S A.

[53] Bossi L, Fuentes JA, Mora G, Figueroa-Bossi N (2003). Prophage contribution to bacterial population dynamics. J Bacteriol.

[54] Davies EV, James CE, Kukavica-Ibrulj I, Levesque RC, Brockhurst MA, Winstanley C (2016). Temperate phages enhance pathogen fitness in chronic lung infection. ISME J.

[55] Gama JA, Reis AM, Domingues I, Mendes-Soares H, Matos AM, Dionisio F (2013). Temperate bacterial viruses as double-edged swords in bacterial warfare. PLoS One.

[56] Sassone-Corsi M, Nuccio SP, Liu H, Hernandez D, Vu CT, Takahashi AA, Edwards RA, Raffatellu M (2016). Microcins mediate competition among Enterobacteriaceae in the inflamed gut. Nature.

[57] Resource Coordinators NCBI (2017). Database resources of the National Center for Biotechnology Information. Nucleic Acids Res.

[58] McNair K, Bailey BA, Edwards RA (2012). PHACTS, a computational approach to classifying the lifestyle of phages. Bioinformatics.

[59] Fouts DE (2006). Phage_Finder: automated identification and classification of prophage regions in complete bacterial genome sequences. Nucleic Acids Res.

[60] Finn RD, Coggill P, Eberhardt RY, Eddy SR, Mistry J, Mitchell AL, Potter SC, Punta M, Qureshi M, Sangrador-Vegas A (2016). The Pfam protein families database: towards a more sustainable future. Nucleic Acids Res.

[61] Fu L, Niu B, Zhu Z, Wu S, Li W (2012). CD-HIT: accelerated for clustering the next-generation sequencing data. Bioinformatics.

[62] Edgar RC (2004). MUSCLE: multiple sequence alignment with high accuracy and high throughput. Nucleic Acids Res.

[63] Mistry J, Finn RD, Eddy SR, Bateman A, Punta M (2013). Challenges in homology search: HMMER3 and convergent evolution of coiled-coil regions. Nucleic Acids Res.

[64] Miele V, Penel S, Duret L (2011). Ultra-fast sequence clustering from similarity networks with SiLiX. BMC Bioinformatics.

[65] Adebali O, Ortega DR, Zhulin IB (2015). CDvist: a webserver for identification and visualization of conserved domains in protein sequences. Bioinformatics.

[66] Remmert M, Biegert A, Hauser A, Soding J (2011). HHblits: lightning-fast iterative protein sequence searching by HMM-HMM alignment. Nat Methods.

[67] Shannon P, Markiel A, Ozier O, Baliga NS, Wang JT, Ramage D, Amin N, Schwikowski B, Ideker T (2003). Cytoscape: a software environment for integrated models of biomolecular interaction networks. Genome Res.

[68] Geer LY, Domrachev M, Lipman DJ, Bryant SH (2002). CDART: protein homology by domain architecture. Genome Res.

[69] Sampson T, Broussard GW, Marinelli LJ, Jacobs-Sera D, Ray M, Ko CC, Russell D, Hendrix RW, Hatfull GF (2009). Mycobacteriophages BPs, Angel and Halo: comparative genomics reveals a novel class of ultra-small mobile genetic elements. Microbiology.

