# Supplementary figures and images for: A widespread family of polymorphic toxins encoded by temperate phages
Source: BMC Biol. 2017 Aug 29;15:75. doi: 10.1186/s12915-017-0415-1 (PMC5576092; doi:10.1186/s12915-017-0415-1)

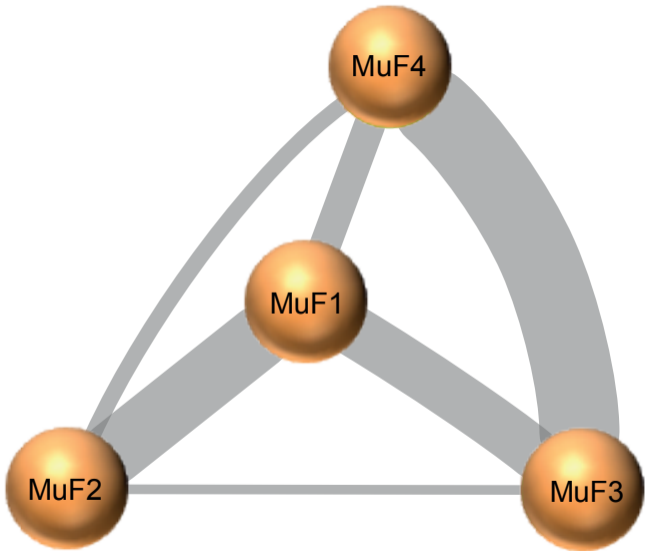

Supplement: Supplementary file 1 — Relationships between MuF families. Network association of the four HMM profiles of MuF proteins. Each node corresponds to a MuF HMM profile, and each edge width is proportional to the probability of homologous relationship computed by HHsearch for pairwise comparison of HMM profiles using HH-suite (see Methods). (PDF 381 kb) [file 12915_2017_415_MOESM1_ESM.pdf]

Length distribution

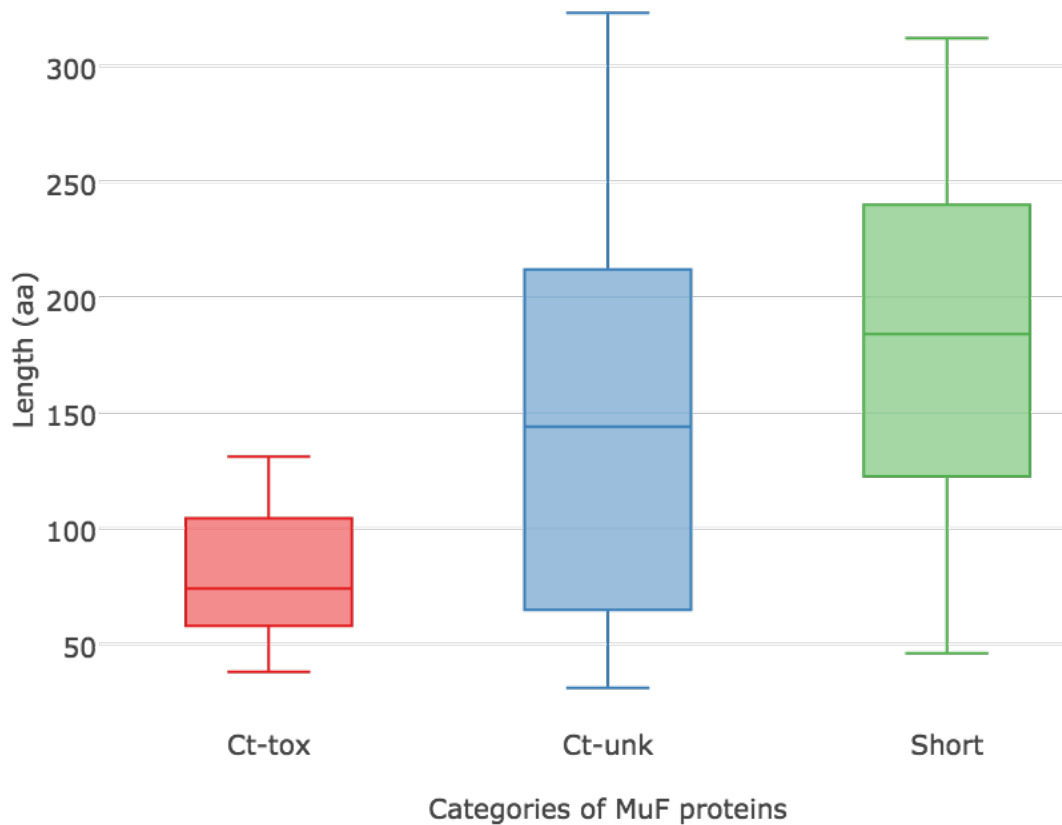

Supplement: Supplementary file 4 — Distribution of the length of the proteins encoded by genes annotated immediately downstream of muf genes. The association of small ORFs with MuF toxins compared to short MuFs is highly significant (two-tailed unpaired Student’s t test, p < 0.0001). The three categories correspond to the MuF protein architecture. Ct-tox MuF domains associated with a C-terminal extension with known toxin domains, Ct-unk MuF domains associated with a C-terminal extension with unknown domains, Short MuF domains without C-terminal extension. (PDF 70 kb) [file 12915_2017_415_MOESM4_ESM.pdf]

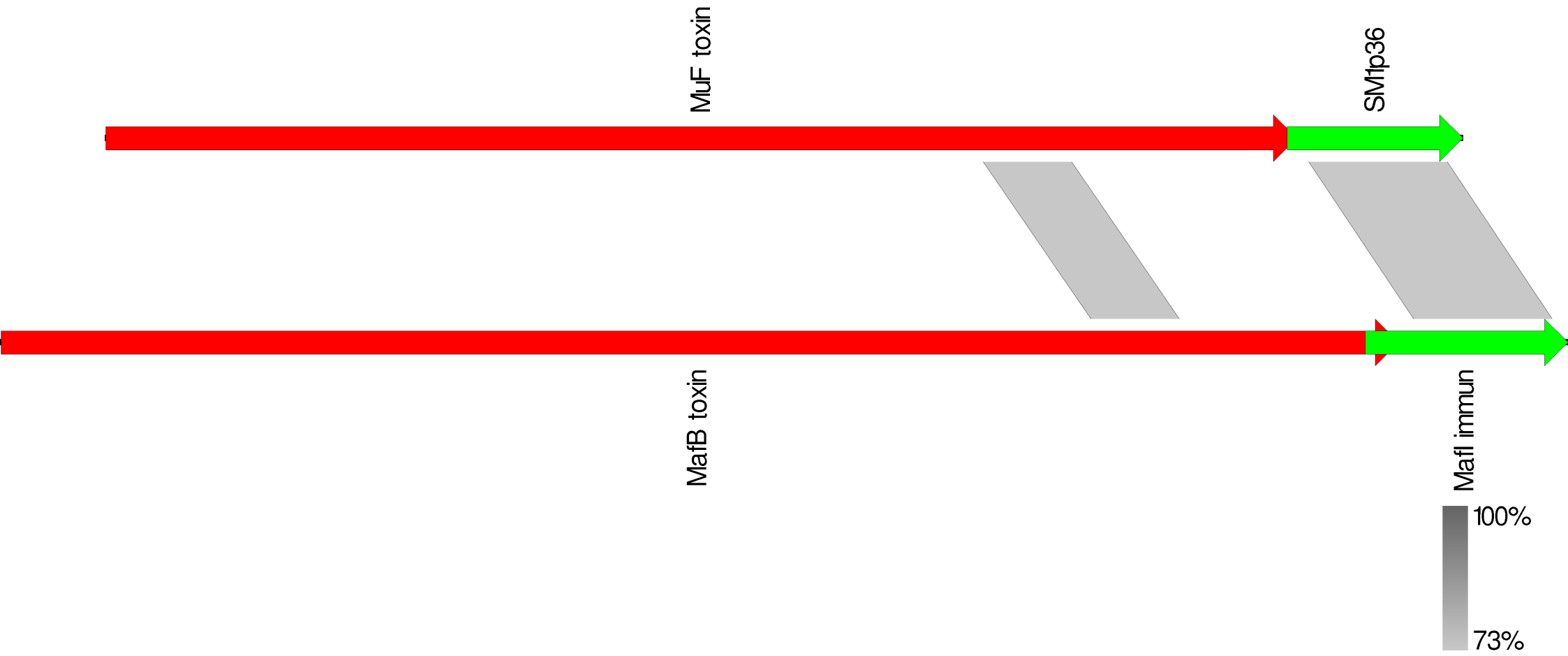

Supplement: Supplementary file 5 — Pairwise comparison of the genetic organization of muf and maf genes. Top: the muf gene and the downstream ORF found in phage SM1 (NC_004996) infecting Streptococcus mitis. Bottom: the mafB toxin gene (encoding WP_003711327.1) and the cognate immunity gene mafI (encoding WP_002235294.1) found in Neisseria meningitidis NM3001 (assembly GCA_000293665.1). Nucleotide comparison was generated using BLASTn implemented in Easyfig 2.1. Gray vertical block indicates regions of shared similarity shaded according to BLASTn identity. The level of nucleotide identity is shown in the gradient scale. (PDF 74 kb) [file 12915_2017_415_MOESM5_ESM.pdf]

A

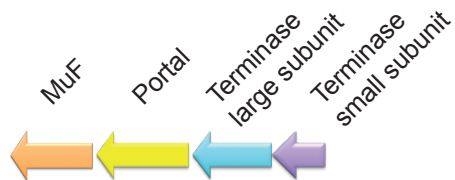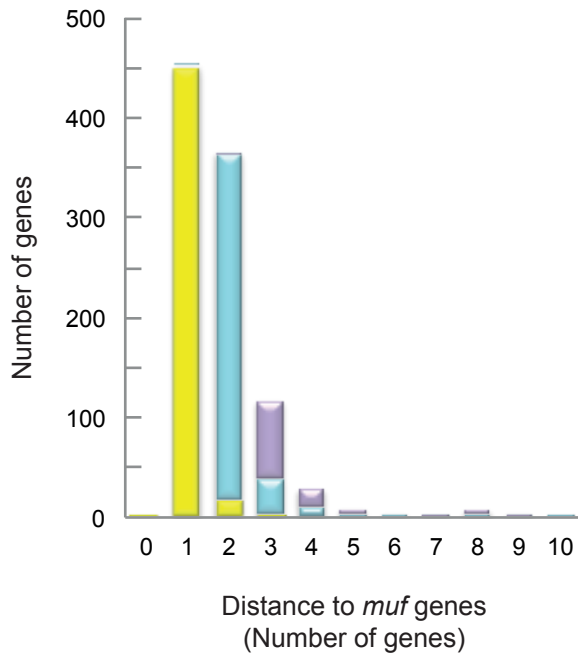

B

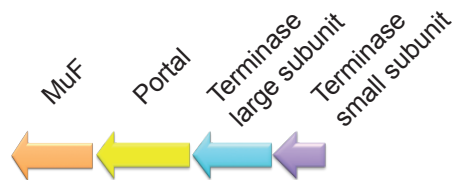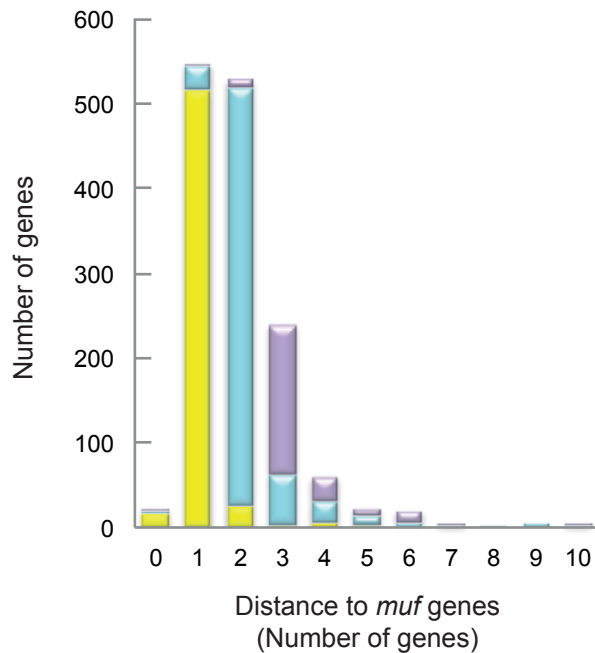

Supplement: Supplementary file 6 — Genetic context in the vicinity of muf genes. Genetic context in the vicinity of muf genes detected in bacteriophage genomes (A) and bacterial chromosomes (typically prophages, B). The total number of genes encoding portal (in yellow), terminase large subunit (in blue), and small subunit (in purple) proteins according to their distances to muf genes (distance = 0) were reported (see Methods). 85% and 87% of muf genes were detected close to at least a portal-encoding gene or a terminase-encoding gene in bacteriophage genomes and bacterial chromosomes, respectively. The genetic context around muf genes was highly conserved in both datasets. (PDF 442 kb) [file 12915_2017_415_MOESM6_ESM.pdf]
