# Supplementary material for: A widespread family of polymorphic toxins encoded by temperate phages
Source: BMC Biol. 2017 Aug 29;15:75. doi: 10.1186/s12915-017-0415-1 (PMC5576092; doi:10.1186/s12915-017-0415-1)

A

MuF protein family

| | MuF1 | MuF2 | MuF3 | MuF4 |
|---|---|---|---|---|
| Proteobacteria | 138 | 0 | 0 | 0 |
| Deferribacteres | - | - | - | - |
| Acidobacteria | - | - | - | - |
| Firmicutes | 121 | 26 | 5 | 0 |
| Tenericutes | 0 | 0 | 0 | 0 |
| Fusobacteria | - | - | - | - |
| Actinobacteria | 48 | 1 | 0 | 267 |
| Cyanobacteria | 2 | 0 | 0 | 0 |
| Chloroflexi | - | - | - | - |
| Spirochaetes | - | - | - | - |
| Bacteroidetes | 5 | 0 | 0 | 0 |
| Chlorobi | - | - | - | - |
| Planctomycetes | - | - | - | - |
| Chlamydiae | - | - | - | - |
| Verrucomicrobia | 0 | 0 | 0 | 0 |
| Aquificae | - | - | - | - |
| Thermotogae | - | - | - | - |
| Deinococcus-Thermus | 1 | 0 | 0 | 0 |
| Synergistetes | - | - | - | - |
| Nitrospirae | - | - | - | - |

C

MuF protein domain

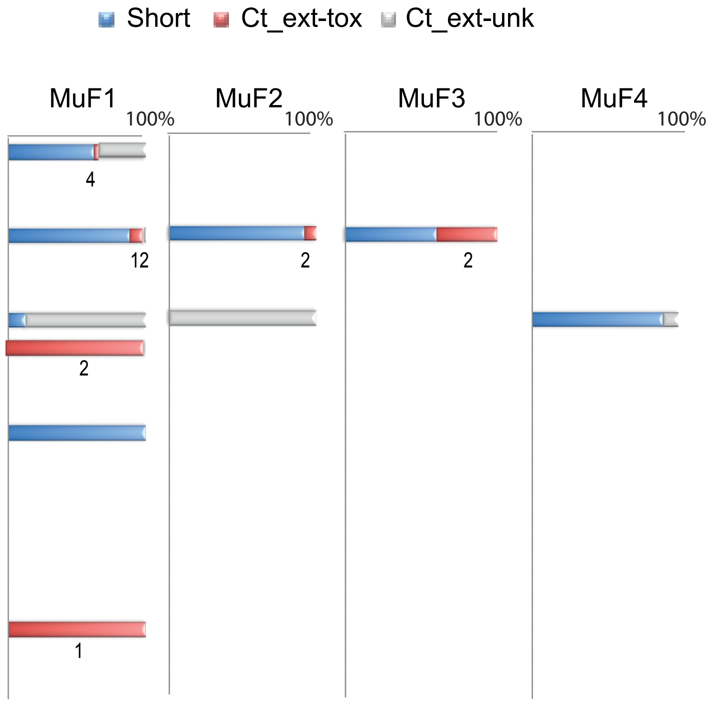


B

MuF protein family

| | MuF1 | MuF2 | MuF3 | MuF4 |
|---|---|---|---|---|
| Proteobacteria | 418 | 0 | 0 | 0 |
| Deferribacteres | 0 | 0 | 0 | 0 |
| Acidobacteria | 1 | 0 | 0 | 0 |
| Firmicutes | 260 | 85 | 21 | 0 |
| Tenericutes | 0 | 0 | 0 | 0 |
| Fusobacteria | 7 | 0 | 0 | 0 |
| Actinobacteria | 6 | 13 | 0 | 48 |
| Cyanobacteria | 2 | 0 | 0 | 0 |
| Chloroflexi | 3 | 0 | 0 | 0 |
| Spirochaetes | 3 | 0 | 0 | 0 |
| Bacteroidetes | 17 | 0 | 0 | 0 |
| Chlorobi | 0 | 0 | 0 | 0 |
| Planctomycetes | 1 | 0 | 0 | 0 |
| Chlamydiae | 0 | 0 | 0 | 0 |
| Verrucomicrobia | 0 | 0 | 0 | 0 |
| Aquificae | 5 | 0 | 0 | 0 |
| Thermotogae | 1 | 0 | 0 | 0 |
| Deinococcus-Thermus | 7 | 0 | 0 | 0 |
| Synergistetes | 0 | 0 | 0 | 0 |
| Nitrospirae | 0 | 0 | 0 | 0 |

clade > 3 genomes

D

MuF protein domain

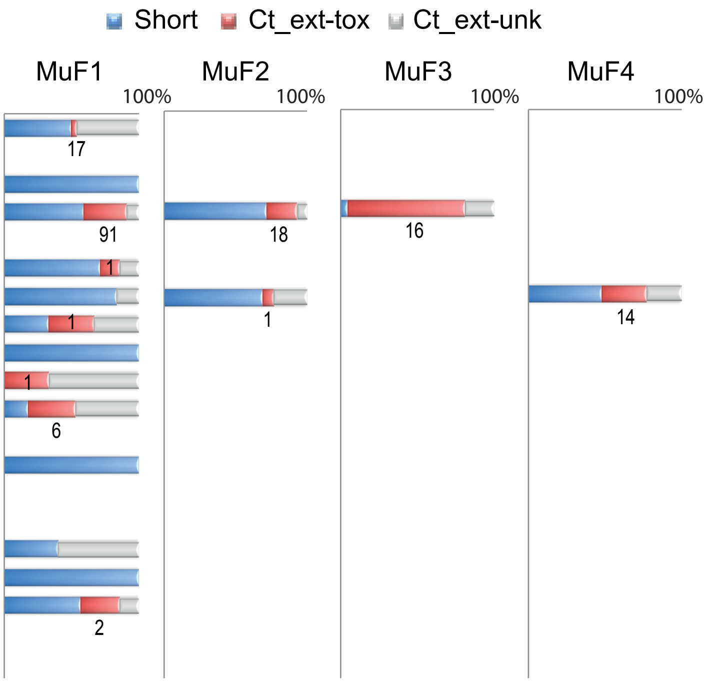

Supplement: Supplementary file 3 — Proportion of toxin domains in each MuF family and taxonomical clade. Distribution of MuF protein families in bacteriophages (A) and in bacterial chromosomes (B). The number of MuF proteins detected was reported (in orange) for each clade. Proportion of MuF domains associated with a C-terminal extension (Ct_ext) with known toxin domains (Ct_ext-tox, in red), with unknown domains (Ct_ext-unk, in gray) or without Ct_ext (Short, in blue) in bacteriophages (C) and in bacterial chromosomes (D). Only the number of MuF proteins with a known toxin domain, and only bacterial clades with at least four genomes sequenced were indicated (for simplicity). (PDF 832 kb) [file 12915_2017_415_MOESM3_ESM.pdf]
